# The Use of Precision Alignment Technology to Circumvent Patient-Specific Roadblocks in Performing Total Knee Arthroplasty: A Case Series

**Published:** 2017-08-30

**Authors:** Thomas J. Hendricks, Alexander CM Chong, Tarun Bhargava

**Affiliations:** 1 University of Kansas School of Medicine-Wichita, Department of Orthopaedics Surgery; 2 Mid-America Orthopedics, Wichita, KS

**Keywords:** total knee arthroplasty, orthopedic procedures, orthopedic devices

## Introduction

The goal of total knee arthroplasty (TKA) is to provide the patient with a well-functioning, pain-free knee that will last for many years. To improve implant survival, the goal is to position the prosthesis in a way that restores proper biomechanical alignment, and advances in technology have enabled arthroplasty surgeons to produce accurate results on a more consistent basis. One of these advances is the use of computer-assisted orthopaedic surgery (CAOS) to assist in proper component alignment and produce accurate restoration of the biomechanical axis consistently.^1^–^17^ Functional scores and revision-free survival are at least equivalent to conventional arthroplasty.^17^–^26^

Traditional cutting guides for TKA rely on intramedullary femoral instruments, and either intramedullary or extramedullary tibial instruments, to obtain proper axial alignment. In cases where retained hardware is present, or in patients with knee osteoarthritis associated with pre-existing femoral or tibial extra-articular deformity, CAOS has proved to be an exceptionally useful, effective, and appealing option.^27^–^37^ A portable, accelerometer-based navigation device (OrthAlign, OrthAlign Inc., Aliso Viejo, CA) for TKA has demonstrated promising results with regard to the alignment accuracy of large-console CAOS systems.^10^–^14^

We present three patients who underwent TKA with retained femoral hardware. Two patients had intramedullary femoral fixation and one patient had an interference screw from a previous anterior cruciate ligament (ACL) reconstruction that would interfere with the use of a conventional intramedullary alignment guide. A novel, portable, accelerometer-based navigation (OrthAlign^®^ precision alignment system, OrthAlign Inc., Aliso Viejo, CA) guide enabled performance of TKA without the need for surgical removal of hardware.

## Case Reports

### Case #1

**Figure 1:**
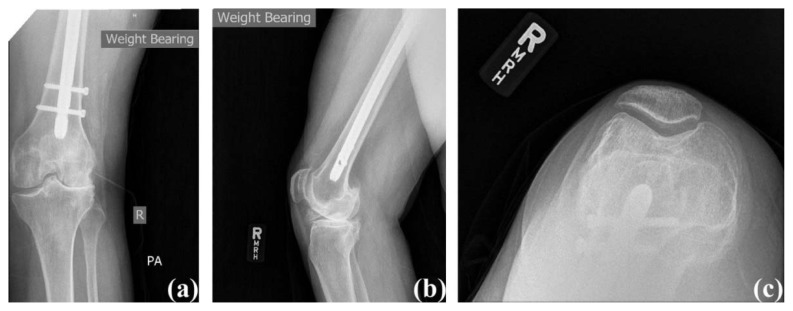
Case #1 pre-operative radiographs: (a) anterior posterior view, (b) lateral view, and (c) sunrise view.

A 52-year-old female (Body Mass Index (BMI): 35.1 kg/m^2^) presented with a chief complaint of right knee pain (Knee Society Score: 57 and functional Knee Society Score: 50). She had a remote history of right intertrochanteric hip fracture treated surgically with a long antegrade cephalomedullary fixation nail. Her fracture proceeded to union, and she was able to bear full weight on the affected extremity. Since her previous surgery, however, she developed anterior and lateral right knee pain accompanied by crepitation. Rest, ice, heat, nonsteroidal anti-inflammatory drugs (NSAIDs) and acetaminophen were used to control her pain but were not successful. Physical examination of her right knee showed correctable valgus deformity and tenderness to palpation both anteriorly and laterally. Her active range of motion was from 0° to 110° of flexion, and she had a mild effusion with crepitus throughout a range of motion. Radiographs showed patellofemoral and lateral compartment joint space narrowing, subchondral sclerosis, and osteophyte formation (Figure 1). The presence of a long antegrade cephalomedullary nail with two distal interlocking screws also was noted.

Given the presence of the long antegrade cephalomedullary fixation nail, a decision was made to use the OrthAlign^®^ precision alignment system to assist in positioning the bone cuts to obviate the need to extract the femoral hardware prior to TKA. During surgery, a standard midline incision was used with a medial parapatellar arthrotomy. Surgery proceeded as usual with the addition of the use of the OrthAlign^®^ precision alignment system to determine the femoral and tibial cuts. The Movation™ system (DJO, LLC, Vista, CA), a posterior stabilized knee system, with femoral size 4, tibia base plate size 2, patella size 32, and an 11 mm posterior, stabilized polyethylene insert was used. Palacios bone cement with vancomycin also was used. Surgical course was without complication. Total tourniquet time was 41 minutes at 300 mmHg.

**Figure 2:**
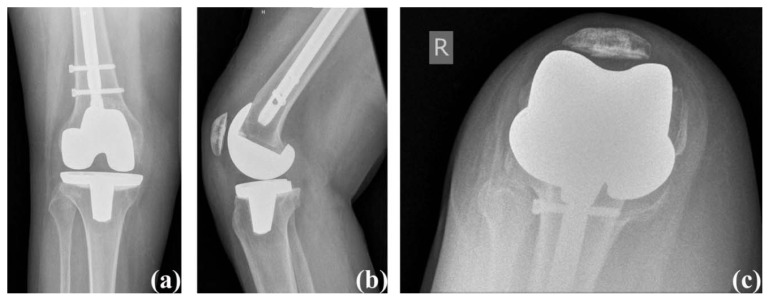
Case #1 post-operative radiographs: (a) anterior posterior view, (b) lateral view, and (c) sunrise view.

Her post-operative course was without complication. She was mobilized and ambulated with physical therapy. She was discharged home on post-operative day two with a prescription for outpatient physical therapy. At six weeks post-operatively, her incision had healed without any complications, her passive range of motion was 0° – 125° and was pain free (Knee Society Score: 100 and functional Knee Society Score: 85). Her knee was stable on exam and well-aligned, radiographically (Figure 2). She was walking unlimited distances with a cane, and she was using stairs in a reciprocal manner. Her next follow-up visit was scheduled for six months after surgery.

### Case #2

**Figure 3:**
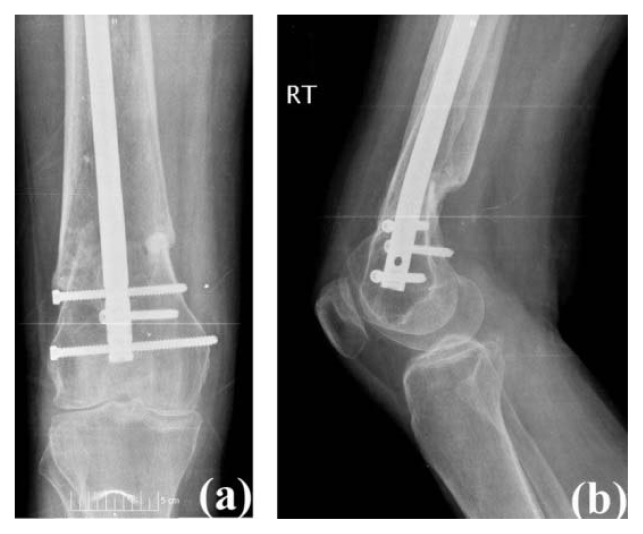
Case #2 pre-operative radiographs: (a) anterior posterior view and (b) lateral view.

A 62-year-old female (BMI: 20.8 kg/m^2^) presented with a chief complaint of right knee pain (Knee Society Score: 52 and functional Knee Society Score: −10). She was a poly-trauma victim, suffering a boating accident ten months prior. She sustained numerous fractures including the spine, pelvis, femur, ankle, and clavicle which required open reduction and internal fixation. Her right femur was treated with a retrograde femoral nail with three distal interlocking screws. Despite the treatment, her right knee pain progressively had been worsening, and was associated with crepitus throughout her range of motion. The use of stairs, as well as prolonged standing and walking, exacerbated her pain. Her pain had not been relieved with conservative treatment measures, or corticosteroid injections. She also noted some discomfort over the two lateral-to-medial distal interlocking screws and requested that these be removed during surgery. Physical examinations revealed a correctable varus deformity of the right knee and active range of motion was from 10° to 105°, accompanied by crepitation. A moderate effusion also was noted along with tenderness along the medial joint line. Radiographs showed a healed fracture of the distal femur at the metaphyseal-diaphyseal junction, and a retrograde femoral nail is visualized with two proximal and three distal interlocking screws (Figure 3).

The presence of the retrograde femoral nail precluded the use of a conventional intramedullary alignment guide. Therefore, navigation-assisted TKA was performed. The two symptomatic distal interlocking screws were removed percutaneously. The Donjoy_©_ surgical foundation cruciate retaining (CR) knee system with femoral size 6, tibia base plate size 4, patella size 32, and an 11 mm standard CR polyethylene insert was used. Surgical course was without complication. Total tourniquet time was 48 minutes at 300 mmHg.

**Figure 4:**
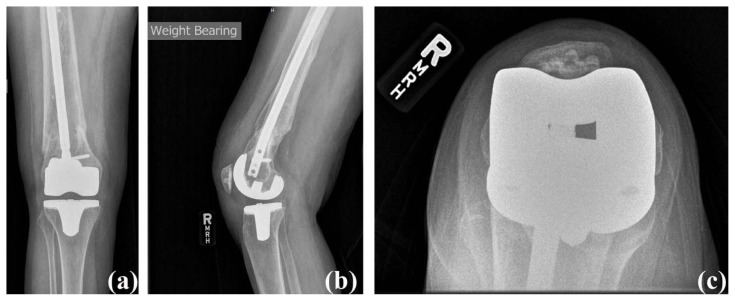
Case #2 post-operative radiographs: (a) anterior posterior view, (b) lateral view, and (c) sunrise view.

The patient was discharged to skilled nursing on post-operative day two for continued physical therapy for approximately two weeks after surgery. She was dismissed home with a prescription for outpatient physical therapy. At six weeks post-operatively, her active range of motion was from 0° to 125° and pain free (Knee Society Score: 100 and functional Knee Society Score: 90). Radiographs showed well-aligned components and restored mechanical axis (Figure 4). She was walking unlimited distances without any assistive device and using stairs in a reciprocal manner with a rail. Her follow-up visit was scheduled for six months after surgery.

### Case #3

**Figure 5:**
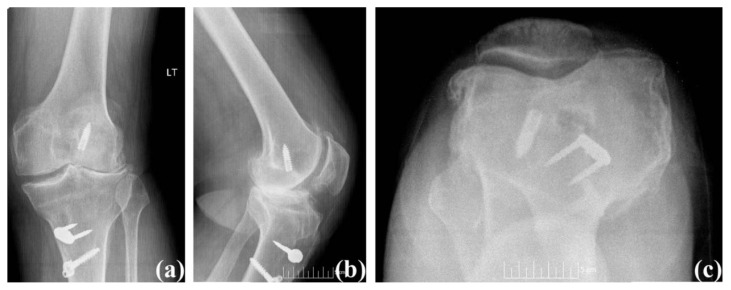
Case #3 pre-operative radiograph: (a) anterior posterior view, (b) lateral view, and (c) sunrise view.

A 60-year-old male (BMI: 31.0 kg/m^2^) presented with a chief complaint of left knee pain. The patient sustained an ACL rupture while playing sports 20 years prior and subsequently underwent an ACL reconstruction. He presented with anterior and lateral left knee pain that was dull, aching, and throbbing, and it progressively had been worsening (Knee Society Score: 56 and functional Knee Society Score: 50). His pain had not been relieved with conservative treatment measures, or corticosteroid injections. Physical examination revealed a 5° valgus deformity with range of motion from 5° to 120° of flexion accompanied by crepitation on lateral and patellofemoral compartment, as well as tenderness to palpation. He was limited to ambulating two to five blocks at a time, and the use of stairs as well as prolonged standing and walking exacerbated the pain. Radiographs showed loss of lateral and patellofemoral compartment joint space with significant sclerosis, osteophyte formation, and retained screws in both the femur and tibia as well as a staple in the tibia from previous ACL reconstruction (Figure 5).

During surgery, it was determined that the tibial screw and staple would need to be removed for placement of the tibial component, and removal of the screw and staple were incorporated as part of the patient’s standard midline incision and medial parapatellar arthrotomy. Given the presence of the femoral interference screw, the navigation-assisted system was used to assist in making accurate bony cuts, orient the implants, assess the soft tissue balancing, and to obviate the need for the screw removal prior to TKA. A posterior stabilized implant was employed, and after making the box cut on the femoral side, the interference screw was protruding about 1 cm into the femoral box; therefore, it was removed. The Movation™ system (DJO, LLC, Vista, CA), a posterior stabilized system with femoral size 8, tibia base plate size 8, patella size 35, and a 9 mm posterior stabilized polyethylene insert and 14 mm tibial modular stem were used. The remainder of the surgical course was without complication. Total tourniquet time was 63 minutes at 300 mmHg.

**Figure 6:**
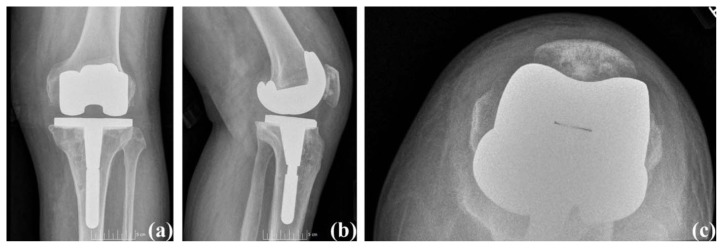
Case #3 Post-Operative Radiographs. (a) Anterior posterior view, (b) Lateral view, and (c) Sunrise view.

At six weeks post-operatively, his knee range of motion was from 0° to 130° of flexion and pain free (Knee Society Score: 100 and functional Knee Society Score: 90). Radiographs showed well-aligned components and restored mechanical axis (Figure 6). He was walking unlimited distances without any assistive device, and he was using stairs in a reciprocal manner with a rail. His follow-up visit was scheduled for six months after surgery.

## Discussion

CAOS can be an effective tool to align TKA component and produce accurate restoration of the biomechanical axis consistently for cases when traditional instrumentation is not possible or appropriate due to posttraumatic femoral deformity, retained femoral hardware, a history of osteomyelitis, or severe cardiopulmonary disease.^27^–^37^ In this report, the rationale for the use of navigation-assisted systems in these three cases was appropriate because they allowed establishment of the biomechanical axis and did not require hardware removal. The use of navigation-assisted system had no adverse impact on the patient’s total time in the operating room or tourniquet time. The post-operative course was not adversely affected, and all three patients’ pain and function were improved at six-week follow-up.

Based on these results, navigation-assisted systems present physicians with an effective option for performing TKA in patients with pre-existing hardware. It obviated the need for a prior surgery to remove the retained implant, saving the patient the risk and subsequent morbidity of a second surgery and conceivably improving patient rehabilitation and outcomes. Furthermore, it also reduced the cost to the patient and the health care system through decreased number of surgeries and total operating room time. Although long-term follow-up was not available, the postoperative course was uneventful. Further research using randomized, prospective studies would be beneficial to compare outcomes in single-stage TKA using navigation-assisted versus two-stage surgery with hardware removal.

In summary, navigation-assisted TKAs with retained femoral hardware were successful and safe. The advantages of the navigated-assisted TKA for normal cases remain a matter of debate, however, navigation-assisted TKA was an excellent alternative for hardware retaining cases.
